# The RNA Chaperone Hfq Impacts Growth, Metabolism and Production of Virulence Factors in *Yersinia enterocolitica*

**DOI:** 10.1371/journal.pone.0086113

**Published:** 2014-01-15

**Authors:** Tamara Kakoschke, Sara Kakoschke, Giuseppe Magistro, Sören Schubert, Marc Borath, Jürgen Heesemann, Ombeline Rossier

**Affiliations:** 1 Max von Pettenkofer Institute for Hygiene and Medical Microbiology, Ludwig Maximilians University, Munich, Germany; 2 Protein Analysis Unit, Adolf-Butenandt Institute, Ludwig Maximilians University, Munich, Germany; University of Osnabrueck, Germany

## Abstract

To adapt to changes in environmental conditions, bacteria regulate their gene expression at the transcriptional but also at the post-transcriptional level, e.g. by small RNAs (sRNAs) which modulate mRNA stability and translation. The conserved RNA chaperone Hfq mediates the interaction of many sRNAs with their target mRNAs, thereby playing a global role in fine-tuning protein production.

In this study, we investigated the significance of Hfq for the enteropathogen *Yersina enterocolitica* serotype O:8. Hfq facilitated optimal growth in complex and minimal media. Our comparative protein analysis of parental and *hfq*-negative strains suggested that Hfq promotes lipid metabolism and transport, cell redox homeostasis, mRNA translation and ATP synthesis, and negatively affects carbon and nitrogen metabolism, transport of siderophore and peptides and tRNA synthesis. Accordingly, biochemical tests indicated that Hfq represses ornithine decarboxylase activity, indole production and utilization of glucose, mannitol, inositol and 1,2-propanediol. Moreover, Hfq repressed production of the siderophore yersiniabactin and its outer membrane receptor FyuA. In contrast, *hfq* mutants exhibited reduced urease production. Finally, strains lacking *hfq* were more susceptible to acidic pH and oxidative stress. Unlike previous reports in other Gram-negative bacteria, Hfq was dispensable for type III secretion encoded by the virulence plasmid.

Using a chromosomally encoded FLAG-tagged Hfq, we observed increased production of Hfq-FLAG in late exponential and stationary phases. Overall, Hfq has a profound effect on metabolism, resistance to stress and modulates the production of two virulence factors in *Y*. *enterocolitica*, namely urease and yersiniabactin.

## Introduction

The genus *Y* *ersinia* includes three human pathogenic species, namely *Y*. *pestis*, the agent of plague and two enteropathogenic species, *Y*. *pseudotuberculosis* and *Y*. *enterocolitica*. We study the Gram-negative bacterium *Y*. *enterocolitica* as a model for an extracellular enteropathogen. Upon ingestion of contaminated food or water, *Y*. *enterocolitica* is able to invade the intestinal submucosa and preferentially multiplies extracellularly in Peyer’s patches and mesenteric lymph nodes [1], [2]. *Y*. *enterocolitica* virulence factors include proteins important for early stages of infection, such as urease, a multisubunit metalloenzyme which facilitates survival to stomach acidity [3], [4] or the outer membrane adhesin called invasin which promotes transcytosis across the epithelial barrier [5]. Two other major virulence factors, which are essential for later stages of infection, are encoded by the virulence plasmid pYV: the outer membrane adhesin YadA and the type III secretion system Ysc (Ysc-T3SS). The Ysc-T3SS is a complex machinery that translocates at least 6 anti-host effector proteins into the host cell (YopH, YopM, YopO, YopT, YopP and YopE), where they collectively inhibit phagocytosis and dampen the inflammatory response [6], [7]. In addition to the pathogenicity factors mentioned above, strains of *Y*. *enterocolitica* biogroup 1B, which are highly virulent in a mouse model of infection, carry a so-called high pathogenicity island (HPI). The HPI encodes proteins involved in production and import of the siderophore yersiniabactin [8]. These proteins include the transcriptional activator YbtA, the biosynthetic enzymes Irp1-Irp5 and Irp9, the inner membrane ABC transporters Irp6 and Irp7, and the yersiniabactin receptor FyuA, which is localized in the outer membrane [8]–[15]. FyuA also confers sensitivity to the bacteriocin pesticin [11]. Importantly, yersiniabactin production and utilization is an essential virulence trait for *Y*. *enterocolitica* in mouse infection [10], [11], [16].

Genes involved in pathogenicity of enteropathogenic *Y* *ersinia* ssp. are regulated by environmental factors such as temperature, ionic strength, pH and host cell contact. For example, under *in vitro* conditions, urease and invasin are most highly expressed at 27°C, the optimal growth temperature [17], [18]. In contrast, pYV plasmid genes encoding the Yop proteins, Ysc-T3SS and the adhesin YadA are upregulated at 37°C, the temperature of the mammalian host [18], [19]. Many transcriptional and post-translational processes underlying pathogenicity gene regulation have been uncovered in *Y* *ersinia* ssp. [18]–[20], but the importance of post-transcriptional mechanisms has only recently become a focus of interest with the discovery of numerous small RNAs (sRNAs) in bacteria. sRNAs (also known as non-coding RNAs) are usually between 50-300 nucleotide long and modulate mRNA translation and/or stability by complementary base-pairing [21]. One key co-factor for many sRNA-mRNA interactions is the RNA chaperone Hfq [22]. Originally described in *Escherichia coli* as a host factor important for the replication of phage Q<beta>, Hfq is an abundant 11-kDa protein that forms hexameric rings. Present in many but not all bacteria, Hfq promotes sRNA-mRNA pairing and sRNA stability. Moreover, it may regulate the activity of proteins involved in mRNA turnover such as RNase E, polynucleotide phosphorylase and poly(A) polymerase and thus may control the stability of numerous gene transcripts. Finally, it is also believed that Hfq might play additional roles in transcription antitermination and translation [22]–[24]. The influence of Hfq on bacterial physiology and virulence has been studied in a growing number of bacterial Gram-negative and Gram-positive pathogens [25], including pathogenic yersiniae [26]–[29]. Hfq generally modulates motility and promotes resistance to stresses likely encountered in the host, such as oxidative stress or low pH [25]. Moreover, it was reported to modulate T3SS in *Salmonella enterica* sv. Typhimurium, enterohemorrhagic *E. coli*, *Vibrio cholerae*, *Pseudomonas aeruginosa* and *Y*. *pseudotuberculosis* [28], [30], [31]. Recent studies have shown distinct phenotypes associated with loss of *hfq* between *Y*. *pseudotuberculosis* and *Y*. *pestis*, with a stronger effect on bacterial growth and on sRNA instability in *Y*. *pestis* [27], [28], [32], [33]. The finding that more of 40% of the sRNAs described in *Y*. *pseudotuberculosis* are not conserved in *Y*. *enterocolitica* [32] suggests that Hfq could modulate protein production differently in the two enteropathogenic *Y* *ersinia* ssp.

A first description of *hfq* in *Y*. *enterocolitica* serotype O:9 (low virulence in mouse infection model) was published in 1996 and described the isolation of spontaneous *hfq* mutants that lost expression of the heat stable enterotoxin Y-ST [26]. In light of the emerging role of Hfq in post-transcriptional gene regulation, we revisited the significance of Hfq for the *Y*. *enterocolitica*, using the highly mouse-virulent strains of serotype O:8. In this study we generated and characterized mutants deleted in the *hfq* gene in two prototypes of *Y*. *enterocolitica* serotype O:8, i.e. WA-314 and the 8081-derivative JB580v. We show that Hfq plays a role in growth, metabolism of carbohydrates and nitrogen, and the production of urease and yersiniabactin siderophore.

## Materials and Methods

### Bacterial strains and media

*Y*. *enterocolitica* and *E. coli* strains used in this study are listed in Table 1. *Salmonella* Typhimurium SB300 and *E. coli* MG1655 were used as positive and negative controls for 1,2-propanediol (1,2-PD) utilization, respectively [34], [35].

**Table 1:** Strains used in this study.

Strains	Description	Source or Reference
*Y*. *enterocolitica*		
WA-314	Clinical isolate of serotype O:8, carrying virulence plasmid pYVO8	[93]
WA-C	pYVO8-cured derivative of WA-314	[93]
WA^RS^	WA-C derivative carrying a deletion in the YenI restriction modification system	[94]
WA-314 pYV-515	WA-314 derivative carrying pYVO8 *lcrD*::Tn5, defective in Yop secretion	[95]
WA *fyuA*	WA-C derivative carrying a nonsense mutation in *fyuA*	[11]
WA *ybtA*	WA-C derivative with the insertion of a Km^R^ cassette inactivating *ybtA*	[96]
SOR3	WA-314 derivative with a deletion of *hfq* marked with a Km^R^ cassette	This study
SOR4	WA-314 derivative with an unmarked deletion of *hfq*	This study
SOR5	pYVO8-cured WA-314 derivative with an unmarked deletion of *hfq*	This study
SOR33	WA^RS^ strain with an unmarked chromosomal fusion of *hfq* with sequences encoding the 3xFLAG epitope	This study
JB580v	Derivative of clinical isolate 8081, restriction endonuclease-negative (R^−^), methyltransferase-positive (M+), carrying virulence plasmid pYVO8	[97]
SOR17	JB580v derivative with a deletion of *hfq* marked with a Km^R^ cassette	This study
SOR35	JB580v derivative with an unmarked chromosomal fusion of *hfq* with sequences encoding the 3xFLAG epitope	This study
8081-U-GB	R^−^M^+^ derivative of clinical isolate 8081, *yeuA*::Km, urease-negative	[4]
*E*. *coli*		
DH5α		[98]
CC118λpir		[99]
*S*. *enterica* serotype Typhimurium		
WR1542	reporter strain WR1330 (*fepA*::Tn*10d*Tc, *iroN*::pGP704, *cir*::MudJ) carrying plasmid pACYC5.2L with genes promoting the import of yersiniabactin (*fyuA*, *irp6-8*), their transcriptional activator (*ybtA*) and a fusion of the *fyuA* promoter to luciferase	W. Rabsch, Wernigerode

*Y*. *enterocolitica* and *E*. *coli* were routinely grown in LB broth (10 g tryptone, 5 g yeast extract and 5 g NaCl per liter) and on LB-agar at 27°C and 37°C, respectively. When indicated yersiniae were also grown on selective yersinia agar (CIN plates, Oxoid, Wesel, Germany), in brain heart infusion (BHI) (Becton Dickinson, Heidelberg, Germany), RPMI 1640 (Invitrogen) and M9 minimal medium [36] supplemented with 0.1% Casamino Acids (Difco, Becton Dickinson), 0.05 µg/ml thiamine and either 1% glucose or 1% glycerol. Finally, to assess sugar utilization by *Y*. *enterocolitica*, we monitored acidification of the agar media around bacterial spots in the presence of neutral red or phenol red. MacConkey agar (Oxoid) as well as LB agar containing 0.003% neutral red were supplemented with either no sugar, glucose (2%), mannitol (2%) or 1,2-PD and vitamin B12 (1% and 200 ng/ml, respectively). Antibiotics were used at the following concentration: ampicillin (Ap) for *E*. *coli*, 100 µg/ml; carbenicillin (Cb) for *Y*. *enterocolitica*, 300 µg/ml; chloramphenicol (Cm), 20 µg/ml; kanamycin (Km), 50 µg/ml; spectinomycin (Sp), 50 µg/ml.

### Growth and metabolic assays

Growth in liquid medium was assessed by measuring the optical density (OD) of the culture at 600 nm over 24 h (Ultrospec 3100 pro spectrophotometer; Amersham Biosciences). Bacterial strains were first precultured overnight in broth at 27 °C, then diluted to OD(600 nm) = 0.1 in 20 ml media and subcultured in Erlenmeyer 125-ml flasks. Bacterial cultures were incubated with shaking at 180 rpm in a Certomat BS-1 incubator (B-Braun Biotech International, Sartorius, Göttingen) at 27 or 37°C.

To observe utilization of sugars, bacteria were either streaked or spotted on media containing a carbohydrate source and pH indicator dye. Overnight cultures in LB were washed and subsequently resuspended in sterile phosphate buffered saline (PBS) to an OD(600 nm) of 0.1. Five microliters were spotted on agar plates, and subsequently incubated at 27°C. Acidification of the medium was observed by the formation of a red or yellow halo around the spots on media containing neutral red or phenol red, respectively. We also analyzed strains with the API-20E biochemical characterization kit (bioMérieux) according to manufacturer’s instructions.

To measure indole production, we used a protocol adapted from Chant and Summers [37]. 0.5 ml bacterial culture supernatant was thoroughly mixed with 0.5 ml HCl-amyl alcohol mixture (75 ml HCl and 225 ml amyl alcohol). After formation of two phases, 0.2 ml of the upper phase was mixed with 1 ml HCl-amyl alcohol mixture containing 5 g/l of 4-dimethylamino-benzalaldehyde (Kovacs’ reagent, Carl Roth). The absorbance of the solution was measured spectrophotometrically at 540 nm and indole concentration was calculated using a standard curve.

### Mutant generation and complementation analysis

To facilitate construction of *hfq* mutants, we used the gene inactivation technique described by Datsenko and Wanner [38]. Primers used are listed in Table 2. Using pACYC177 [39] as a template and primers OR1 and OR2, we amplified by PCR a DNA fragment encoding a Km resistance (Km^R^) cassette flanked on the one side by the *hfq* start codon and 47 bp upstream, and, on the other side, by the *hfq* stop codon and 47 bp downstream. To eliminate the template plasmid, the PCR product was subsequently digested with *Dpn*I and precipitated. To express the λ phage Red recombination functions in *Y* *ersinia*, strains WA-314 and JB580v harbouring plasmid pKD46 were grown in LB supplemented with 1% arabinose and subsequently made electrocompetent [36]. Following electroporation of the PCR product, recombinant bacteria were selected with Km and further analysed by PCR with primers OR14 and OR15 to confirm correct allelic exchange. The *hfq*-negative strains thus derived from WA-314 and JB580v were designated SOR3 and SOR17, respectively. Loss of pKD46 and maintenance of the virulence plasmid pYV was confirmed by PCR using primer pairs OR20/OR21 and OR33/OR34, respectively. In these mutants the Km^R^ cassette might have a polar effect on the expression of *hflX*, the gene downstream of *hfq*.

**Table 2:** Primers used in this study.

Primer name	Sequence
OR1hfqKanfor	CGATAGGTTCTTAGTTAATAACAACAAGCAAATAAGGAAAATATAGAATGTCACTGACACCCTCATCAGTG
OR2hfqKanrev	TGAATCCGTTGCTTATGTTCCCCGTCATGGTTGACCAGCAATGCGCTTTACGTCAAGTCAGCGTAATGCTC
OR5hfqp1	AGCCGATAGGTTCTTAGTTAATAACAACAAGCAAATAAGGAAAATATAGAGTGTAGGCTGGAGCTGCTTC
OR6hfqp2	GCCTGAATCCGTTGCTTATGTTCCCCGTCATGGTTGACCAGCAATGCGCTCATATGAATATCCTCCTTAG
OR8hfqHindIII	AACATAAGCTTGAATCCGTTGC
OR11hfqFLAG	GTAATCCATCTGCGCCGCAACAGCCGCAGCAGGATAGCGATGACGCTGAAGACTACAAAGACCATGACGG
OR14hfq	GGTTGCGGGGCTGGGGTTCA
OR15hfq	GTAGTCGCAAAGCACCGCACCCT
OR20pKD46beta	CCTTTCCTGATAAGCAGAATG
OR21pKD46beta	AATCCAAGAGCTTTTACTGC
OR30hfqUSSalI	TGATGTCGACGAAATGGTTTACC
OR33yscCfor	ACCGCGAAACCTTATGTCAC
OR34yscCrev	AAACCCTACTTCCAGACAAG
OR35pcp20ApF	GCGATCTGTCTATTTCGTTC
OR36pcp20ApR	ACCAGTCACAGAAAAGCATC

We also generated a mutant with an unmarked deletion of *hfq*. Here primers OR5 and OR6 were used along with template plasmid pKD3 to amplify a Cm^R^ cassette flanked on the one side by the *hfq* start codon and 47 bp upstream, and, on the other side, by the *hfq* stop codon and 47 bp downstream. Two Cm^R^ colonies were selected from two independent electroporations of strain WA-314(pKD46). Correct insertion of the Cm^R^ cassette was verified by PCR. Following growth at 37°C, the strains were subsequently cured of the plasmid pKD46 and electroporated with helper plasmid pCP20. To express the FLP recombinase strains harbouring pCP20 were grown overnight at 37°C in LB and subsequently plated on LB agar. Cm-sensitive colonies were further analysed by PCR for the loss of the resistance cassette, loss of pCP20 and maintenance of the pYV plasmid. Two independent *hfq* deletion mutants were thus derived from WA-314, i.e. SOR4 and SOR27.

To assist in the detection of Hfq, we tagged the chromosomal gene with a sequence encoding the 3xFLAG epitope using the method described by Uzzau et al. [40]. Using plasmid pSUB11 as a template and primers OR11 and OR6, we amplified a Km^R^ cassette flanked by sequences encoding the last 16 aminoacids of Hfq fused to the 3xFLAG epitope on one side and on the other side, 47 bp downstream of the *hfq* gene. Km^R^ colonies were isolated after electroporation of WA^RS^(pKD46) or JB580v(pKD46). Elimination of the Km^R^ was carried out with pCP20 as described above. Km-sensitive colonies were then analysed by PCR for the loss of the resistance cassette, loss of pCP20 and maintenance of the pYV plasmid. Strains SOR33 and SOR35, derived from strains WA^RS^ and JB580v, respectively, were selected for further analysis.

For complementation analysis, a 635–bp DNA fragment containing *hfq* with 266–bp upstream sequence was amplified using primers OR30 and OR8 and genomic DNA from JB580v as a template, digested with *Sal*I and *Hin*dIII and cloned into pACYC184 (New England Biolabs) to generate pAhfq. To clone *hfqFlag* into pACYC184, we used the same strategy as for pAhfq except that SOR35 genomic DNA was used for the PCR, thus producing pAhfqFlag. To inactivate the tetracycline resistance cassette of plasmid pACYC184 we digested the pACYC184 with *Bam*HI and *Sal*I, generated blunt ends fragments with Klenow fragment of DNA polymerase I and religated the vector, thereby generating pACYC184ts.

### Subcellular protein fractionation, two-dimensional gel electrophoresis (2-DE)

Bacteria were grown overnight at 27°C, subsequently diluted in fresh LB to an OD(600 nm) of 0.1, and then grown in triplicate at 37°C for 5 h (100 ml culture in 500 ml-Erlenmeyer flasks). Cells were collected from 50 ml culture by centrifugation at 4°C for 15 min at 4300 x rpm (Centrifuge 4K15, rotor 12169-H, Sigma). Bacterial cell pellets were resuspended in 1/5 of the original volume with buffer (40 mM sodium phosphate, 50 mM NaCl, pH 7.8, 4 mM phenylmethanesulfonyl fluoride) and cells were then disrupted by two passages in a French Press. Cell lysates were then centrifuged at 4°C for 30 minutes at 18,170 x g to separate soluble proteins (in supernatant) from total membrane proteins (in pellet). One milliliter of soluble proteins was precipitated overnight at –20°C with an equal volume of 20% TCA diluted in acetone. Following centrifugation, precipitated protein pellets were washed twice with acetone, air dried and resuspended in 250 µl of 2DE-loading buffer {8 M urea, 2% 3-[(3-cholamidopropyl)dimethylammonio]-1-propanesulfonate (CHAPS), 1% dithiothreitol (DTT), 2% Pharmalyte pH 3–10 carrier ampholytes (GE Healthcare)}. Pellets of total membrane proteins were resuspended in 1 ml of 10% TCA in acetone, incubated overnight at –20°C and subsequently centrifuged as above. Pellets were washed twice in acetone, air dried and resuspended in 300 µl of 2-DE loading buffer. Protein concentrations were determined using the Bradford protein assay kit (Bio-Rad) according to the manufacturer’s instructions, using BSA as a standard. To further confirm that proteins amounts were comparable, protein samples were loaded on standard SDS-PAGE gels, followed by Coomassie blue staining. pH 3–10 strips (NL, 7 cm, Bio-Rad) were rehydrated for 12 h with 200 µg proteins in rehydration buffer (8 M urea, 2% CHAPS, 0.4% DTT, 0.5% Pharmalyte) in the isoelectric focusing cell (Protean IEF cell, Bio-Rad) set at 50 V. Isoelectric focusing was done for a total of ∼8,000 V-hr. For the second-dimension separation, strips were first equilibrated for 10 min in 2% DTT in equilibration buffer (6 M urea, 2% SDS, 50 mM Tris pH 8.8, 20% glycerol) followed by a 10-min incubation in 2.5% iodoacetamide in equilibration buffer, and were then loaded onto 12.5% SDS-PAGE gels. Gels were run in a Dodeca cell (Bio-Rad), stained with Coomassie blue and scanned under high resolution using the GS-800 calibrated densitometer (Bio-Rad). For this analysis, three biological replicates for each strain were compared. Data were then analysed using the PDQuest 2-D analysis software from Bio-Rad v. 8.0.1, and included spot matching, normalization and intensity averaging. Spots for which the intensity ratio wt/*hfq* mutant was either ≥2 or ≤0.5 were excised and further processed for mass spectrometry (except spot 3601, identified as AtpD, for which the ratio was 1.79).

### Trypsin digest and mass spectrometry

In-gel digests were performed as described in standard protocols. Briefly, following the SDS-PAGE and washing of the excised gel slices proteins were reduced by adding 10 mM DTT (Sigma Aldrich) prior to alkylation with 55 mM iodoacetamide (Sigma Aldrich). After washing and shrinking of the gel pieces with 100% acetonitrile, trypsin (Sequencing Grade Modified, Promega) was added and proteins were digested overnight in 40 mM ammoniumbicarbonate at 37°C.

For protein identification, we used MALDI or LC-MS/MS. For MALDI, 10 µl of each sample were first purified and concentrated on a C18 reversed phase pipette tip (ZipTip, Millipore) prior to elution of the peptides with 1 µl of <alpha>-cyano-4-hydroxycinnamic acid (HCCA, Sigma) and directly spotting on a MALDI sample plate (Applied Biosystems). MALDI-TOF measurements were then performed on a Voyager-DE STR Time Of Flight (TOF) mass spectrometer (Applied Biosystems). Alternatively, protein identification probes were directly used for nano-ESI-LC-MS/MS. Each sample was first separated on a C18 reversed phase column via a linear acetonitrile gradient (UltiMate 3000 system, Dionex) and column (75 µm i.d. x 15 cm, packed with C18 PepMap™, 3 µm, 100 Å, LC Packings) before MS and MS/MS spectra were recorded on an Oribitrap mass spectrometer (Thermo Scientific). The resulting spectra were analyzed via the Mascot™ Software (Matrix Science) using the NCBInr Protein Database.

### Protein staining and immunoblotting

Protein extracts from comparable OD(600 nm) equivalents were denatured at 95–100°C for 5 min, chilled on ice, and separated by SDS-PAGE (Mini-Protean Tetra-cell, Bio-Rad, Munich). Gels were stained by Coomassie blue staining [41] or silver staining using the PageSilver kit (Fermentas, St.Leon-Rot, Germany). For immunoblotting, proteins separated by SDS-PAGE were transferred to PVDF membranes using a Semi-Dry-Blotter (Carl Roth, Karlsruhe) according to manufacturer’s instructions. Following overnight blocking in 3% skim milk diluted in PBS, membranes were reacted with primary antibodies diluted in PBS with 0.1% Tween 20 (PBST) for 1 h at room temperature (RT), washed three times for 10 min in PBST, and incubated for 1 h at RT with PBST containing the secondary antibody conjugated to horseradish peroxidase (HP) (GE Healthcare, Freiburg). After three 5-min washes in PBST, chemiluminescence detection was performed using the Amersham ECL Western blotting analysis system (GE Healthcare) and membranes were exposed to an X-ray film (Fujifilm superRX, Hartenstein). Developed films were scanned with the GS-800 calibrated densitometer (Bio-Rad) and Western blot semi-quantification was performed with QuantityOne software (v. 4.5.0, Bio-Rad). In each experiment, loading of equivalent amounts of proteins was controlled by Coomassie blue staining and/or by equivalent amounts of cross-reacting bands in immunoblots (when applicable).

Primary antibodies used were rabbit antisera directed against UreB [4] (1:1,000 dilution, a kind gift from S. Batsby, Freiburg), FyuA [10], YopB, YopD, LcrV [42], YopP, YopE, YopM [43], YopH [44], and YopQ [45] (all antibodies specific for Yop proteins were diluted 1:5,000). We also used mouse monoclonal antibodies directed against the FLAG epitope of tagged proteins (anti-FLAG M2, 1:2000, Sigma). Secondary antibodies were HP-conjugated anti-rabbit or anti-mouse immunoglobulin G (GE Healthcare), both diluted 1:10,000.

### Sensitivity assays

To measure survival to acid or oxidative stress, overnight cultures in LB were diluted in PBS (pH 7.5) to 10^7^ CFU/ml. 0.5 ml bacterial suspension was mixed with an equal volume of PBS acidified with acetic acid to pH 4.0 (acid stress), or PBS containing 1 mM H_2_O_2_ (oxidative stress) or PBS at pH 7.5 (mock-treated). Bacteria were incubated at 37°C for 90 min and then bacterial dilutions were plated to determine CFUs. Percentage survival is defined as the CFUs after treatment x 100/CFUs after mock-treatment.

Minimal inhibitory concentrations of antibiotics were determined on LB agar using M.I.C.Evaluator strips (Oxoid).

Sensitivity to pesticin was assessed as previously described [10].

### Yersiniabactin detection

*Y*. *enterocolitica* strains were cultivated in LB medium supplemented with 0.2 mM α,α’-dipyridyl (DIP) for 24 h at 37°C (Fur-derepressed conditions). Bacteria were pelleted by centrifugation and their supernatant was added to the *Salmonella* siderophore indicator strain WR1542 carrying plasmid pACYC5.3L (kind gift of W. Rabsch, Wernigerode). The plasmid encodes all genes necessary for yersiniabactin uptake (*irp6*, *irp7*, *irp8*, *fyuA*) and *ybtA*. Additionally, the *fyuA* promoter region fused to the luciferase reporter gene *luc* is included on pACYC5.3L. The indicator strain was grown in presence of bacterial supernatants for 24 h at 37°C, after which it was centrifuged and lysed with bacterial lysis buffer (100 mM potassium phosphate buffer [pH 7.8], 2 mM EDTA, 1% [wt/vol] Triton X-100, 5 mg/ml bovine serum albumin, 1 mM dithiothreitol, 5 mg/ml lysozyme). Complete lysis was performed by incubation at room temperature for 20 min and repeated mixing. The samples were centrifuged and supernatants were tested by addition of luciferase reagent (20 mM Tricine-HCl (pH 7.8), 1.07 mM (MgCO_3_)_4_Mg(OH)_2_, 100 µM EDTA, 470 µM D(–) luciferin, 33.3 mM dithiothreitol, 270 µM Li_3_ coenzyme A, 530 µM Mg-ATP). Luciferase activities were determined in triplicates using the multimode reader Tristar LB 941 (Berthold Technologies, Bad Wildbad, Germany). Values were normalized to the OD(600 nm) of the *Y*. *enterocolitica* bacterial cultures. *E. coli* strain DH5α served as negative control.

### Type III secretion assay

For studying Yop secreted proteins released into culture supernatant by the Ysc-T3SS, bacteria were first precultured overnight in LB (or BHI) at 27°C, diluted in 20 ml LB (or BHI) to OD(600 nm)  = 0.1 and incubated for 90 min at 37°C. Then, to induce Yop secretion, we added MgCl_2_ and ethylene glycol tetraacetic acid (EGTA) to a final concentration of 10 mM and 5 mM, respectively [46]. After incubation for 90 min at 37°C, the cultures were centrifuged at 2,600 x g for 10 min at 4°C. The supernatants were further cleared by passing through a 0.2 µm low protein binding filter and then precipitated overnight with 0.1 volume trichloroacetic acid (TCA) on ice. Following centrifugation at 10,000 x g for 30 min at 4°C, the pellet was resuspended in 1 ml PBS with a cell scraper, precipitated with 8 ml freezer-cold (–20°C) acetone for 30 min on ice and centrifuged at 10,000 x g for 30 min at 4°C. A second wash was performed with 1 ml freezer-cold acetone, after which the pellet was dried before resuspension in Laemmli buffer. The volume of Laemmli buffer used was adjusted according to the OD(600 nm) of each culture with the following formula: ODx100 µl. To prepare bacterial lysates, cell pellets from centrifuged cultures were resuspended in ODx200 µl of PBS. Then 100 µl of bacterial suspension was mixed with 100 µl of 2x Laemmli buffer.

## Results

### Deletion of *Y. enterocolitica hfq* gene and its effect on general growth characteristics

Examination of the genome sequence of *Y*. *enterocolitica* strain 8081 showed that it encodes a protein with 82% identity and 88% similarity to *E*. *coli* Hfq. The cluster of genes flanking *hfq* is similar to that described in *Y*. *pestis* and *Y*. *pseudotuberculosis* as well as in *E*. *coli* [29], [47]. As a first step in understanding the significance of post-transcriptional regulation for *Y* *. enterocolitica*, we used allelic exchange to replace the entire *hfq* coding sequence by a Km^R^ cassette in two strains of *Y*. *enterocolitica* serotype O:8 of different lineages, i.e. WA-314 and 8081-derived JB580v [48], [49]. Mutant strains SOR3 and SOR17 were isolated from strains WA-314 and JB580v, respectively. In addition, we also generated an independent unmarked deletion mutant in strain WA-314, i.e. *hfq*-negative strain SOR4.

**Figure 1:**
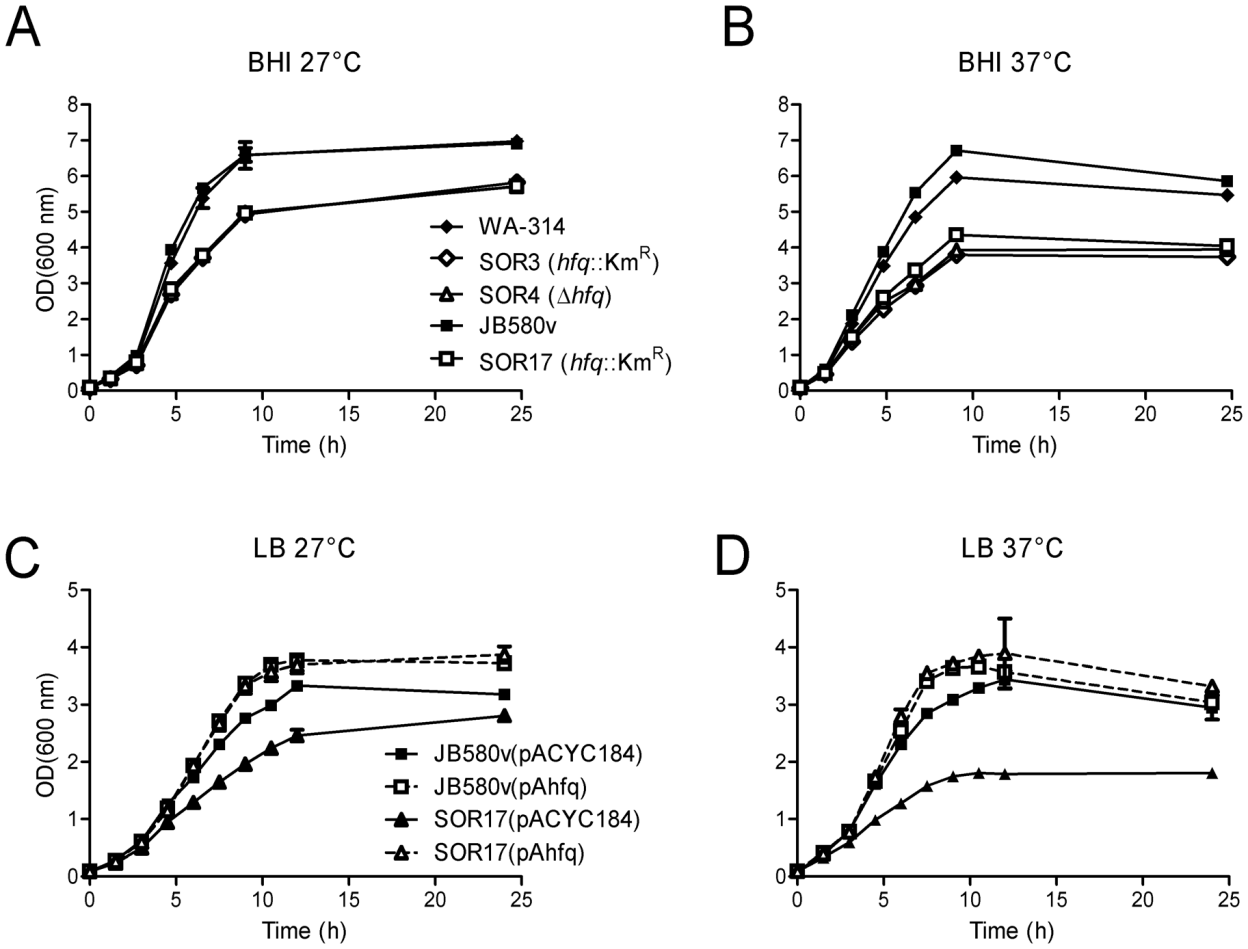
Growth of *Y*. *enterocolitica* strains in BHI (A, B) and LB (C,D). (A and B) Bacteria were grown in BHI at 27°C (A) and 37°C (B): parental strains WA-314 (black diamonds) and JB580v (black squares), *hfq*-negative strains SOR3 (white diamonds), SOR4 (white triangles) and SOR17 (white squares). (C and D) Growth in LB of complemented strains at 27°C (C) and 37°C (D): JB580v(pACYC184) (black squares and straight line, parental strain harbouring the control plasmid), JB580v(pAhfq) (white square and dotted line, parental strain with complementing plasmid), SOR17(pACYC184) (black triangle and black line, *hfq*-negative strain with control plasmid), and SOR17(pAhfq) (white triangle and dotted line, complemented *hfq* strain). Full complementation of the growth defect of strains SOR3 and SOR4 was also observed after introduction of plasmid pAhfq (data not shown). Results are the mean and standard deviation (error bars) of two cultures and are representative of at least two independent experiments.

All *hfq*-negative strains formed normal colonies on LB and BHI agar at 27°C (size, surface, color). However, in LB and BHI liquid media, the *hfq* mutants exhibited a slowed growth rate at 27°C (Fig. 1). Moreover, compared to parental strains, the mutants entered stationary phase at a lower OD, which correlated with the reduction in the CFUs recovered from the broth cultures (Fig. 1 and data not shown). When bacteria were grown at 37°C, the growth rate of the *hfq* mutants was further reduced, as well as the OD reached in stationary phase (Fig. 1). Introduction of *hfq*-containing plasmid pAhfq restored normal growth in all three mutant strains at 27°C and 37°C (Fig. 1). In the course of the complementation experiments, we noted that pAhfq also promoted growth of parental strains WA-314 and JB580v to higher OD upon transition from exponential phase into stationary phase at both temperatures, probably due to an increase in *hfq* copy number (Fig. 1C-D). We also investigated growth of *hfq*-negative strains in minimal M9 medium supplemented with glucose or glycerol (to bypass catabolite repression) at 27°C. With both carbon sources, *hfq* mutants reached stationary phase at a lower OD than wild types (data not shown). Taken together, these results show that the presence of the *hfq* gene promotes optimal bacterial growth in *Y* *. enterocolitica*.

We next examined cell shapes of all bacterial strains by light microscopy. Upon growth in LB at 27°C for 16 h, *hfq*-negative strains were more elongated and slightly wider than parental strains (data not shown). Therefore, lack of Hfq leads to a change in cell morphology of *Y*. *enterocolitica*, as has been described for other bacteria [25], [50], including *Y*. *pestis* [29].

### Proteomic analysis

**Figure 2:**
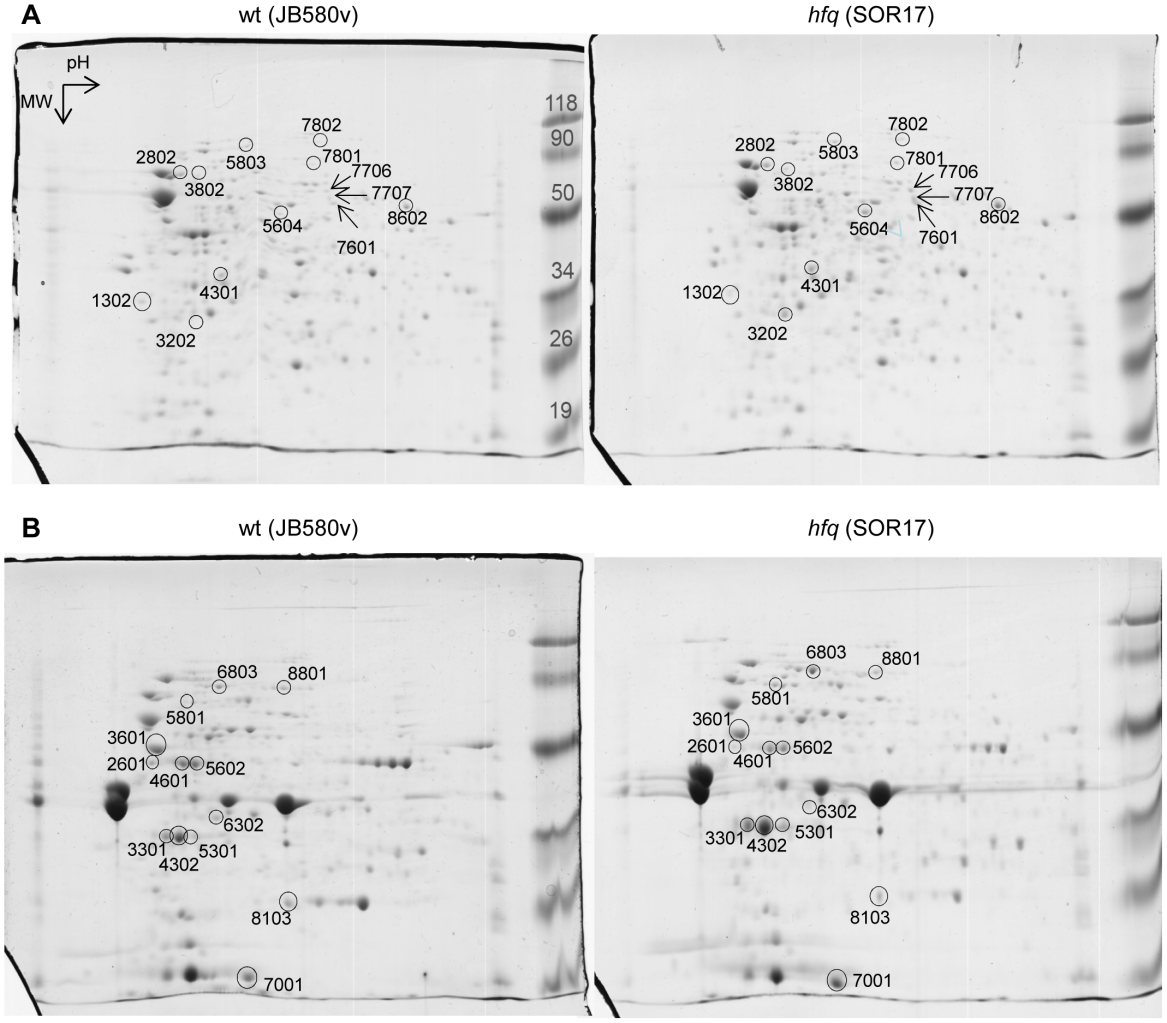
2-DE analysis of total soluble (A) and total membrane (B) proteins stained with Coomassie blue. Bacteria were grown in triplicate at 37°C for 5 h. One representative gel per strain is shown. Proteins were separated in 2-DE gels (for all gels: pH range 3–10, molecular weight (MW) range 15–150 kDa). Highlighted spots were identified by mass spectrometry (see Table 3). MW marker size is indicated in kDa.

To assess the scope of proteins of which production and/or stability are affected by Hfq in *Y*. *enterocolitica*, we used two-dimensional electrophoresis (2-DE) to compare the proteome of strain JB580v with that of the *hfq*-negative derivative SOR17. Bacteria were grown in triplicate in LB for 5 h at 37°C (temperature of the infected host). After French-press treatment, the disrupted cells were separated into total soluble and total membrane protein fractions. Proteins were then subjected to 2-DE and stained with Coomassie blue. In all replicates analyzed, several protein spots showed a reproducible difference in abundance between the protein fractions of the *hfq* mutant and those of the wild type (Fig. 2). Mass spectrometry allowed the identification of proteins from 26 spots, which matched 21 distinct proteins from *Y*. *enterocolitica* strain 8081 (Table 3). In addition, one protein present only in SOR17 (spot 3202) corresponded to the neomycin phosphotransferase encoded by the Km^R^ cassette used to mark the deletion of the *hfq* gene.

**Table 3:** *hfq*-dependent changes in protein abundance found by 2-DE analysis upon growth in LB for 5 h at 37°C.

Spot #	Regulation^a^	MW (kDa)	YE #	Gene name	Protein description	GO Biological process^b^
**Soluble proteins**						
1302	–	32	YE3057	*ybbN*	putative thioredoxin	
2802	+	71	YE3090	*htpG*	heat shock protein 90	protein folding; response to stress
7601, 8602	+	50	YE0741	*degP*/*gsrA*/*htrA*	serine endoprotease	proteolysis
5803	+	96	YE0893	*clpB*/*htpM*	protein disaggregation chaperone	protein metabolic process; response to stress
7802	+	88	YE3132	*lon*/*capR*	DNA-binding ATP-dependent protease La	ATP-dependent proteolysis
3802	+	64	YE3258	*proS*/*drpA*	prolyl-tRNA synthetase	prolyl-tRNA aminoacylation
4301	+	35	YE0604	*tal*/*talB*	transaldolase B	pentose-phosphate shunt
7801	+	72	YE3416	*tkt*/*tkt1*/*tktA*	transketolase	metabolic process (pentose-phosphate)
5604	+	53	YE0650	*tnaA*	tryptophanase	tryptophan catabolic process
7707	+	51	YE2164	*pykF*	pyruvate kinase	glycolysis
7706	+	62	YE2233	*oppA*	periplasmic oligopeptide-binding protein precursor	transport
3202	+	31	N/A^c^	*nptII*	neomycin phosphotransferase [Escherichia coli]	
**Insoluble proteins**						
2601	–	46	YE1274	*fadL/trr*/*todX*	putative long-chain fatty acid transport protein	lipid transport
3601	–	50	YE4206	*atpD*	F0F1 ATP synthase subunit beta	plasma membrane ATP synthesis coupled proton transport
4601, 5602	–	43	YE0278	*tufA*/*tuf1*	elongation factor Tu	translational elongation
6302	–	36	YE3268	*accA*	acetyl-CoA carboxylase carboxyltransferase subunit alpha	fatty acid biosynthetic process
8103	–	22	YE3174	*ahpC*	putative alkyl hydroperoxide reductase subunit c	cell redox homeostasis
8801	–	67	YE0364	*frdA*/*b4154*	fumarate reductase flavoprotein subunit	anaerobic respiration
3301, 4302, 5301	+	35	YE3039	*lpxR*/*sfpA*	lipopolysaccharide deacylase, systemic factor protein A	
5801	+	74	YE2622	*fyuA*	outer membrane pesticin and yersiniabactin receptor	siderophore transport; iron ion transport
6803	+	74	YE1771	*fcuA*	outer membrane ferrichrome receptor protein FcuA	siderophore transport; iron ion transport
7001	+	19	YE2835	*ompX*	outer membrane protein X	

^a^ , +: protein more abundant in *hfq*-negative strain; –: protein less abundant in *hfq*-negative strain; ^b^, Gene ontology biological function used in the GenoList database (http://genodb.pasteur.fr); ^c^, N/A: not applicable.

Proteins less abundant in the *hfq* mutant are likely involved in lipid metabolism and transport (AccA, FadL), cell redox homeostasis (AhpC), modulation of protein chaperones (YbbN), anaerobic respiration (FrdA), translation (TufA), and ATP synthesis (AtpD) (Table 3). Proteins upregulated in the *hfq*-negative strain included chaperones and proteases involved in response to stress (HtpG, ClpB, DegP, Lon), or proteins implicated in carbon metabolic pathways (TalB, TktA, PykF), amino acid catabolism and peptide transport (TnaA, OppA), tRNA synthesis (ProS), as well as four OMPs (LpxR/SfpA, OmpX, and TonB-dependent siderophore receptors FyuA and FcuA) (Table 3).

We also performed a less extensive 2-DE comparison of total protein content of bacteria grown for 16 h at 27°C, with only one replicate per strain. Using mass spectrometry we identified nine spots which were more abundant in the *hfq* mutants (Table 4). Three proteins had previously been identified in the analysis undertaken at 37°C, i.e. FcuA, TnaA and DegP (Tables 3 and 4). Five newly identified proteins were all predicted to play a role in 1,2-propanediol (1,2-PD) utilization (PduA, PduB, PduC, PduD and PduG) (Table 4), a metabolic activity believed to promote adaptation of *S*. Typhimurium and *Listeria monocytogenes* to particular niches in host tissues [51]. The last spot found to be more abundant in the *hfq* mutant is a putative periplasmic binding protein encoded by gene *ye2751*, which flanks the *pdu* region, and, unlike the *pdu* genes, is conserved in *Y*. *pseudotuberculosis* and *Y*. *pestis*. Based on conserved domain CD06302, YE2751 could be involved in the transport of pentose or hexose sugars.

**Table 4:** *hfq*-dependent changes in protein abundance found by 2-DE analysis upon growth in LB for 16 h at 27°C.

Regulation^a^	MW (kDa)	YE #	gene name	Protein description	GO biological function^b^
+	80	YE1771	*fcuA*	ferrichrome receptor protein	siderophore transport
+	50	YE0650	*tnaA*	tryptophanase	tryptophan catabolic process
+	51	YE0741	*degP*/*htrA*/*gsrA*	serine endoprotease	proteolysis
+	38	YE2751		putative periplasmic binding protein	transport
+	70	YE2730	*pduC*/*pddA*	putative propanediol utilization protein: dehydratase, large subunit	metabolic process
+	70	YE2733	*pduG*/*ddrA*	putative propanediol utilization protein: diol dehydratase reactivation	
+	33	YE2731	*pduD*/*pddB*	putative propanediol utilization protein: dehydratase, medium subunit	
+	32	YE2729	*pduB*	putative propanediol utilization protein	response to external stimulus
+	10	YE2728	*pduA*	putative propanediol utilization protein	

^a^ , +: protein more abundant in *hfq*-negative strain; ^b^, Gene ontology biological function used in the GenoList database (http://genodb.pasteur.fr).

Taken together, the results of the 2-DE analysis suggest that Hfq impacts metabolism, surface proteins and stress responses of *Y*. *enterocolitica*.

### Influence of hfq on carbohydrate metabolism

**Figure 3:**
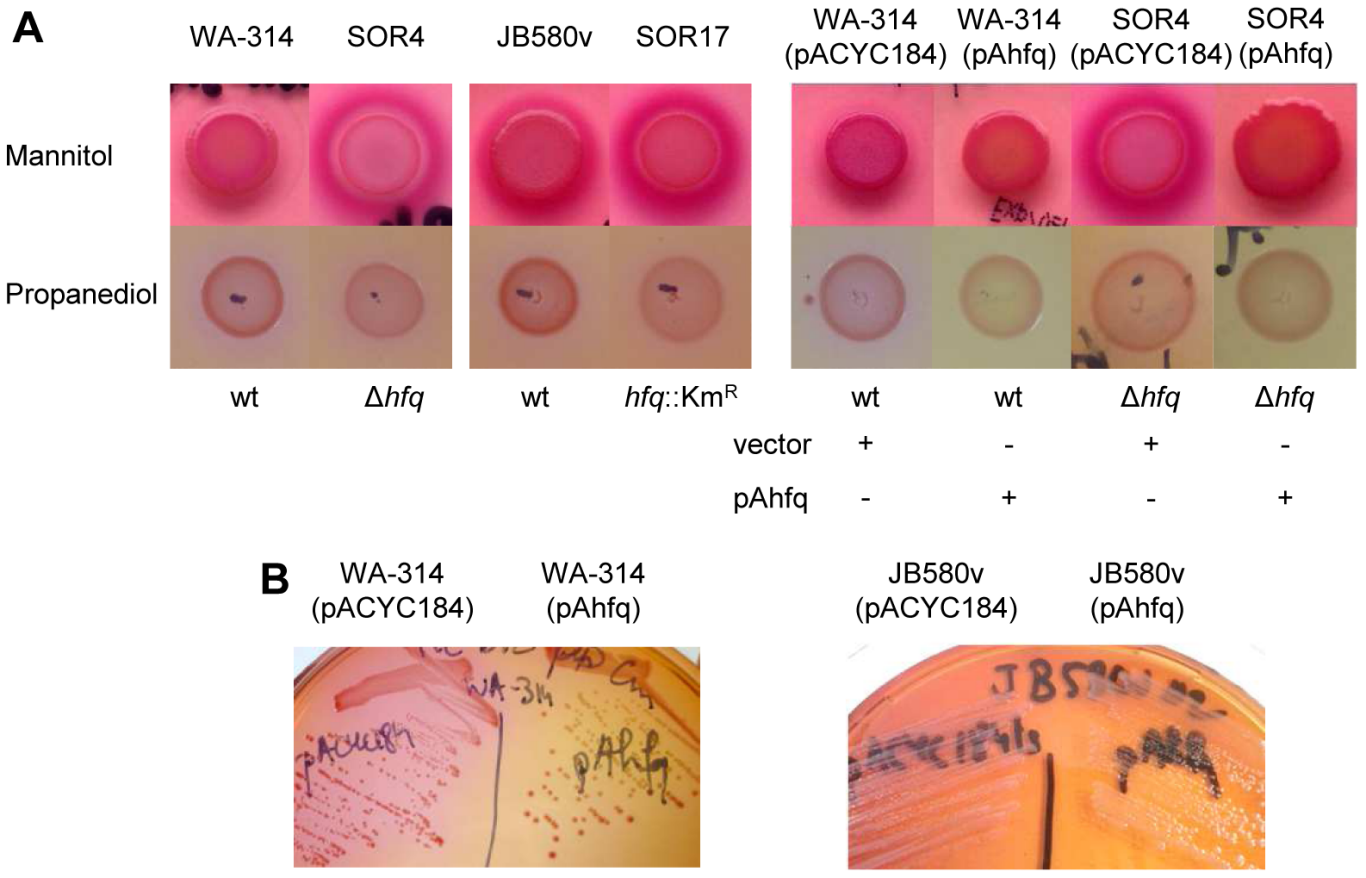
Influence of *hfq* on carbohydrate metabolism. (A) Bacteria were spotted on CIN agar (top row) and MacConkey agar supplemented with vitamin B12 and 1,2-PD (bottom row). Plates were incubated at 27°C for three (top) or two days (bottom). (B) Bacteria were grown on MacConkey agar supplemented with vitamin B12 and 1,2-PD at 27°C for two days.

As the next step in our analysis, we explored the influence of Hfq on carbohydrate metabolism. First, we performed biochemical tests using the API-20E kit. Using a pH indicator dye, this kit detects the release of organic acids upon bacterial growth in the presence of different carbohydrates. Amygdalin was the only carbohydrate for which all *hfq*–negative strains exhibited reduced medium acidification compared to the corresponding parental strains (data not shown). Interestingly, growth in the presence of inositol led to increased medium acidification for *hfq* mutants SOR3 and SOR4 compared to the parental strain WA-314 (data not shown), similarly to what has been described in *Y*. *enterocolitica* O:9 [26]. Strains lacking *hfq* exhibited typical acidification of the media after growth for 24 h at 27 °C in the API-20E wells containing glucose, mannitol, sorbitol, sucrose and arabinose. However, we wondered if the slowed growth of *hfq* mutants could mask differences between parental and mutant strains. Bacterial suspensions inoculated to the API-20E strips are very dilute and an increase in acidification might not be detectable because parental and mutant bacteria are at different stages of growth. A hint that this might be the case came from the following observation: upon growth on *Y* *ersinia* selective agar (CIN agar) for two days at 27 °C, all strains produced colonies with the typical dark pink bull’s eye pattern, indicative of mannitol utilization (mannitol is the only carbohydrate in CIN agar). However, we noticed that all the *hfq*-negative strains also produced a strong pink halo surrounding areas of heavy bacterial growth. Indeed, when we spotted bacterial suspensions on CIN agar, we observed that after two days of incubation at 27°C, spots of *hfq* mutants were surrounded by a sharp dark pink halo that intensified over the next 2 days (Fig. 3A). The wild type also produced a halo but only one or two days later, suggesting that acidification of the agar medium was quicker for the *hfq*-negative strains. Complementation of this phenotype was achieved with plasmid pAhfq (Fig. 3A and data not shown). To confirm that the halo appearance was independent of the dye used to monitor acidification, we also spotted bacterial cultures on an agar medium containing mannitol and phenol red (instead of neutral red): indeed, strain SOR4 produced a yellow halo that appeared earlier and was stronger than the one produced by wild-type WA-314 (data not shown). We also grew bacteria on MacConkey agar supplemented with different sugars. Since *Y*. *enterocolitica* does not utilize lactose, all strains grown on unsupplemented MacConkey produced yellow colonies, whereas plates supplemented with mannitol, glucose or sucrose gave rise to red/pink colonies (data not shown). When bacterial suspensions were spotted on MacConkey agar with mannitol or glucose, the pink halos were stronger for *hfq* mutants SOR4 and SOR17 compared to their parental strains WA-314 and JB580v. On MacConkey agar containing sucrose, we could only observe a very faint pink halo around all the spots with no noticeable differences between parental strains and mutants (data not shown). Finally, we also used MacConkey agar containing 1,2-PD and vitamin B12, an essential co-factor for the Pdu enzyme complex. Similar to medium containing sucrose, acidification around bacterial spots of the wild types was very faint (Fig. 3A). Because *hfq* mutants grew slightly more slowly on this medium, we could not easily compare them to their parental strains (Fig. 3A). However, we noticed that expressing additional copies of *hfq* in the parental strains (from plasmid pAhfq) led to a reduction in the pink color of spots or colonies (Fig. 3A and B), evoking a decrease in 1,2-PD utilization. Overall, our results suggests that Hfq represses the catabolism of mannitol, glucose, inositol and 1,2-PD in *Y*. *enterocolitica*.

### Influence of hfq on nitrogen metabolism

**Figure 4:**
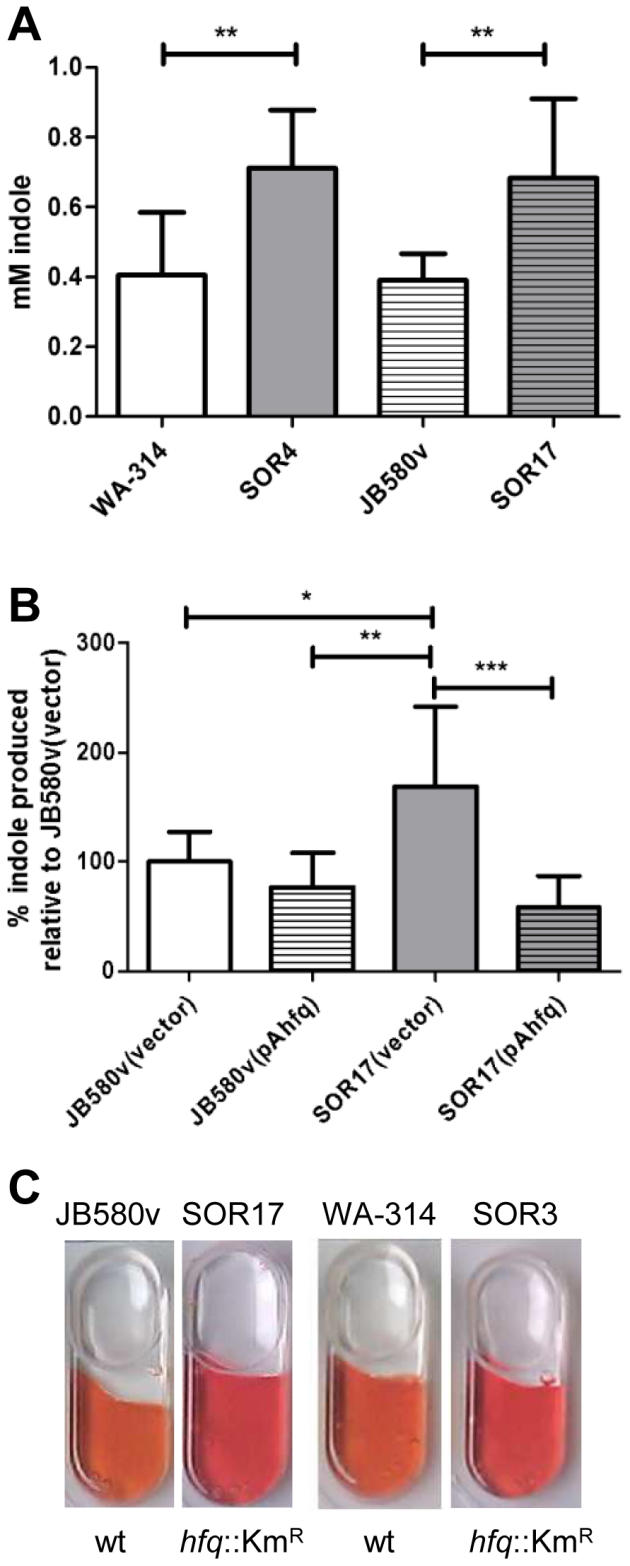
Influence of *hfq* on indole production and ornithine decarboxylase activity. (A and B) The concentration of indole present in culture supernatants was determined after growth in LB at 27°C. (A) Bacteria were grown for four hours at 27°C. Data represent mean and standard deviation of at least three independent experiments each performed with triplicate independent cultures. (B) Complementation analysis. Bacterial cultures were grown for 16 h at 27°C, since strains carrying plasmids were delayed in their indole production. Because of the variability of indole concentration produced by parental strains carrying plasmids (between 0.07 and 1.5 mM in four independent experiments), results were expressed relative to the indole produced by the parental strain JB580v carrying the control vector(which was set at 100%). Data represent mean and standard deviation of four independent experiments each performed with at least triplicate independent cultures. Significance was calculated with Student‘s unpaired *t*-test (*P<0.05; **P<0.01; ***P<0.001). (C) Ornithine decarboxylase activity detected using the API-20E strip. All wells are positive (negative wells remain yellow), but wells inoculated with *hfq*-negative strains turn red, whereas those inoculated with parental strains are more orange.

Next we examined the influence of *hfq* on nitrogen metabolism. Since our 2-DE proteome analysis suggested that tryptophanase was more abundant in a *hfq* mutant, we used Kovacs’ reagent to detect the production of indole, the by-product of tryptophanase activity. Upon growth in LB at 27°C, *hfq* mutants produced more indole than the parental strains, a phenotype that was complemented for bacteria carrying plasmid pAhfq (Fig. 4A and B). Therefore, together with results from 2-DE, our analysis indicates that Hfq represses the production of tryptophanase.

Using the API-20E strips, we observed that the ornithine decarboxylase activity was also markedly increased for all strains lacking *hfq* when bacteria were grown at 27 °C (Fig. 4C), suggesting that polyamine synthesis is also modulated by *hfq*.

**Figure 5:**
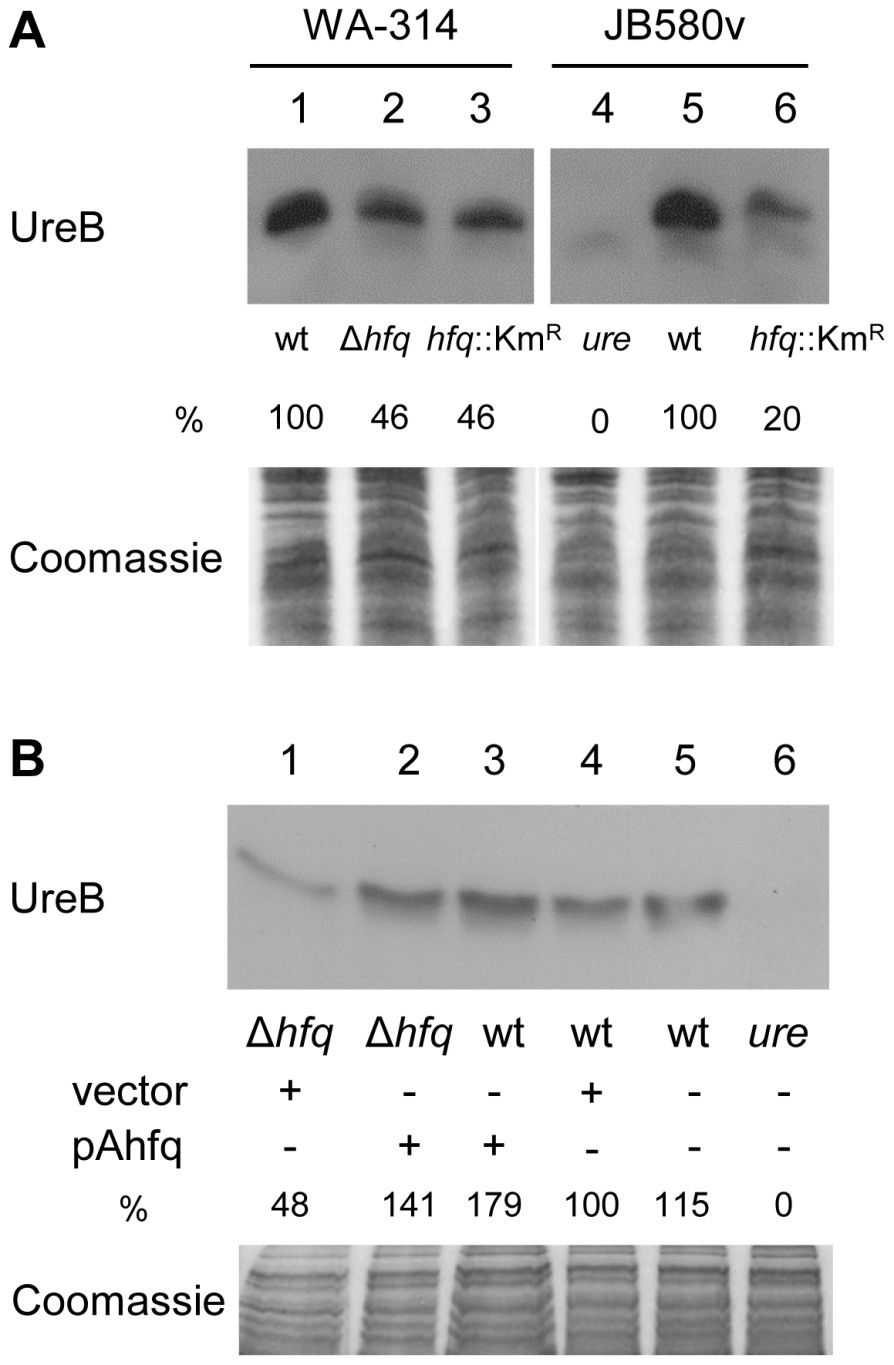
Immunodetection of the 19-kDa urease beta subunit in total protein extracts of *Y*. *enterocolitica*. The relative signal for each band compared to wild type (which was set to 100%) is indicated. Upper panel shows the immunoblot, bottom panel shows part of the Coomassie blue-stained gel used as loading control. (A) Loading was as follows: 1, WA-314; 2, SOR4; 3, SOR3; 4, urease-negative control strain 8081-U-GB; 5, JB580v, and 6, SOR17. (B) Complementation analysis. Loading was as follows: 1, SOR4(pACYC184ts); 2, SOR4(pAhfq); 3, WA-314(pAhfq); 4, WA-314(pACYC184ts); 5, WA-314; and 6, 8081-U-GB. In another experiment, we also observed restoration of the production of UreB in the *hfq*-negative strain SOR17 carrying pAhfq (data not shown).

Finally, we also noted that urease activity was decreased for all *hfq* mutants compared to their corresponding parental strains in the API-20E strips (data not shown). To further assess the influence of *hfq* on the production of urease, we performed immunoblotting using a rabbit polyclonal antibody specific for the 19-kDa UreB subunit [17]. Bacteria were grown overnight at 27°C, conditions described for maximal urease production [3], [4]. Figure 5 shows that the urease production is reduced in *hfq*-negative strains relative to wild types, although the reduction observed in the WA-314 derivatives was more modest than in strain JB580v (50% and 80% reduction, respectively). Complementation was observed after introduction of pAhfq in the mutant strains (Fig. 5B compare lane 1 and 2). Thus, Hfq enhances the production of urease, a known virulence/fitness factor of *Y*. *enterocolitica*.

### Role of hfq in susceptibility to acidic, oxidative and antibiotic stress

**Figure 6:**
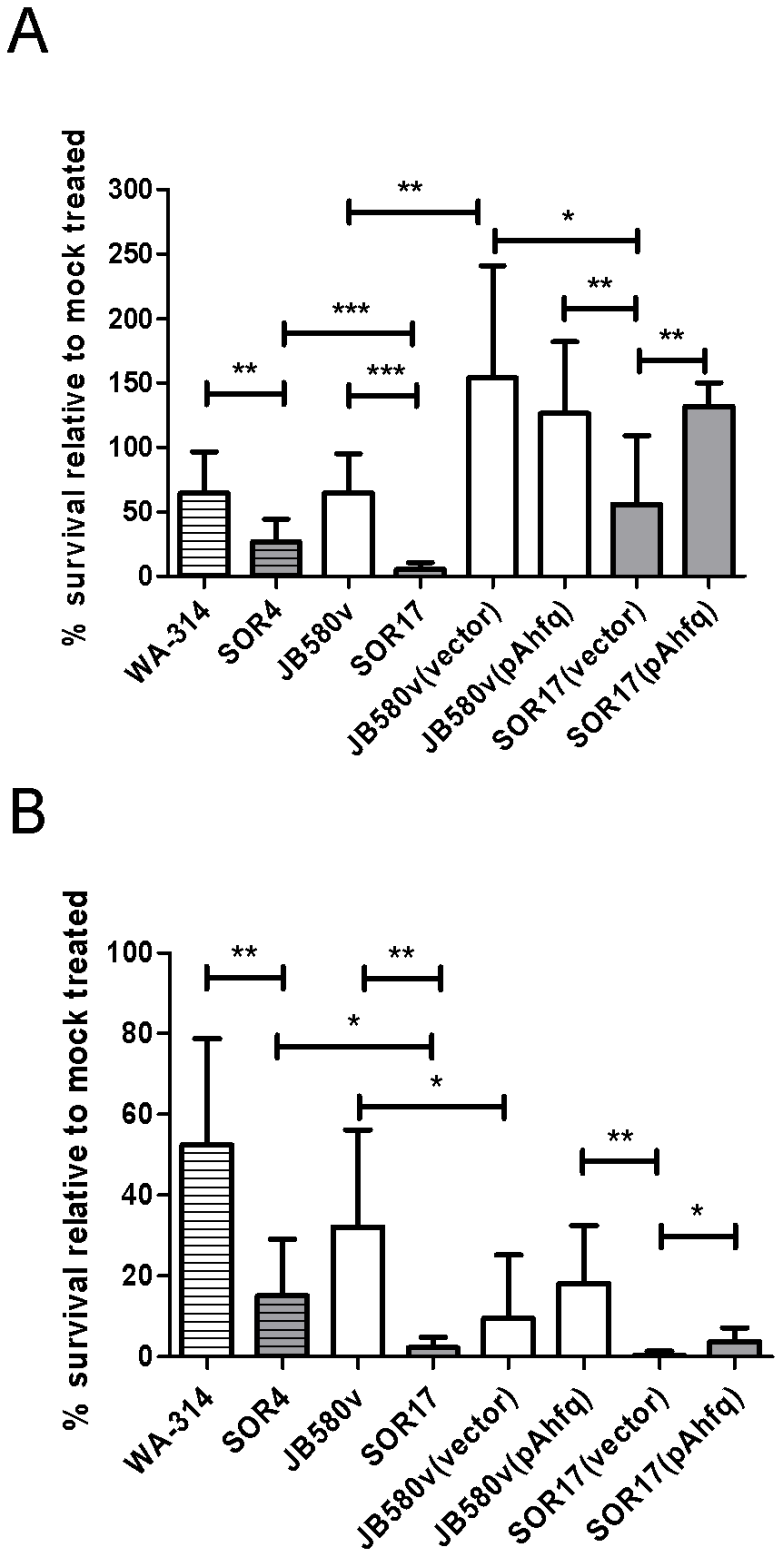
Influence of *hfq* on bacterial survival to acidic and oxidative stress. (A) Bacterial survival to exposure to pH 4.0 for 90 min. (B) Bacterial survival to exposure to 1 mM H_2_O_2_ for 90 min. Results are expressed as % survival relative to bacteria incubated in PBS pH 7.5 and are the mean and standard deviation of at least three experiments performed with three separate cultures. Complementation assays correspond to two independent experiments performed with at least three separate cultures. Significance was calculated with Student‘s unpaired *t*-test (*P<0.05; **P<0.01; ***P<0.001). Bacterial strains are WA-314 and its *hfq*-negative derivative SOR4, JB580v and its *hfq*-negative derivative SOR17.

Since urease is known to contribute to resistance to acidic pH, we tested whether a *hfq*-negative strain would be more susceptible to acid stress using a survival assay. As shown in Fig. 6A, both *hfq*-negative strains SOR4 and SOR17 exhibited a reduced survival at pH 4.0 compared to their parental strains. Mirroring its more pronounced decrease in urease production, strain SOR17 was more susceptible to acidic stress than strain SOR4 (6% compared to 26% respectively). Using plasmid pAhfq, we observed complementation of the survival defect of strain SOR17 (Fig. 6A). Hence, in *Y*. *enterocolitica*, Hfq promotes resistance to acidic stress.

As a next step in our study, we analyzed bacterial susceptibility to additional stress challenges, i.e. oxidative and antibiotic stress. Both *hfq* mutants SOR4 and SOR17 were more susceptible to killing by hydrogen perdoxide that parental strains, with again an even stronger phenotype in strain SOR17 (Fig. 6B).Introduction of the complementation plasmid pAhfq into SOR17 increased the strain’s resistance to H_2_O_2_ (Fig. 6B). Therefore, as described for other bacteria and *Y* *ersinia* species [25], [27], [28], Hfq promotes survival of *Y*. *enterocolitica* in the presence of oxidative stress.

Finally we determined the minimal inhibitory concentration (MIC) of several antibiotics for WA-314 and SOR4: no significant differences in MIC were observed between strains for all antibiotics tested, i.e. ampicillin, oxacillin, gentamicin and trimethoprim/sulfamethoxazole (data not shown). Therefore, lack of *hfq* does not lead to general increase in sensitivity to antibiotics.

### Influence of hfq on production of the siderophore receptor FyuA

Among the OMPs whose production was increased in the *hfq* mutant, the 2-DE proteomic analysis identified FyuA (Table 3), which is an essential virulence factor in *Y*. *enterocolitica* biotype 1B strains [11]. FyuA functions as the receptor for the siderophore yersiniabactin but also for the bacteriocin pesticin [11]. To confirm the influence of Hfq on FyuA production under conditions where iron is not depleted, we performed a pesticin susceptibility assay using a disk diffusion assay (Table 5). As observed previously [11], a strain lacking *fyuA* is resistant to killing by pesticin, as denoted by the absence of growth inhibition even at the highest concentration of pesticin (Table 5). In contrast, the *hfq*-negative strain SOR4 was more susceptible to pesticin compared to the parental strain WA-314: SOR4 showed an increase in both the size of the growth inhibition zone and in the minimum dilution factor required to observe growth inhibition (MID), a phenotype that was complemented by expressing *hfq* from plasmid pAhfq (Table 5). In the course of the complementation experiment, we also noted that pAhfq rendered the wild-type strain WA-314 completely resistant to pesticin (Table 5). Using this assay, we observed some strain differences: strain JB580v appeared more susceptible than strain WA-314 to pesticin. Lack of *hfq* renders JB580v only slightly more susceptible to the bacteriocin with a modest 2-fold increase in the MID (Table 5). In summary, Hfq appears to repress susceptibility to pesticin, which is likely to reflect its influence on the production of FyuA.

**Table 5:** Pesticin sensitivity assay^a^.

Strains	Genotype	Halo diameter^b^ (cm)	MID^c^
WA-314	wt	1.0	2
SOR4	*hfq*	1.3	12
WA *fyuA*	*fyuA*	0	<1
WA-314(pAhfq)	wt (*hfq* ^+^)	0	<1
SOR4(pACYC184)	*hfq* (vector)	1.5	16
SOR4(pAhfq)	*hfq* (*hfq* ^+^)	0.8	1
JB580v	wt	1.2	16
SOR17	*hfq*	1.2	32

^a^ , all strains were tested in duplicate at least twice and a representative experiment is shown; ^b^, size of growth inhibition obtained with undiluted pesticin preparate; ^c^, MID: minimum inhibitory dilution factor for pesticin preparate to inhibit bacterial growth.

**Figure 7:**
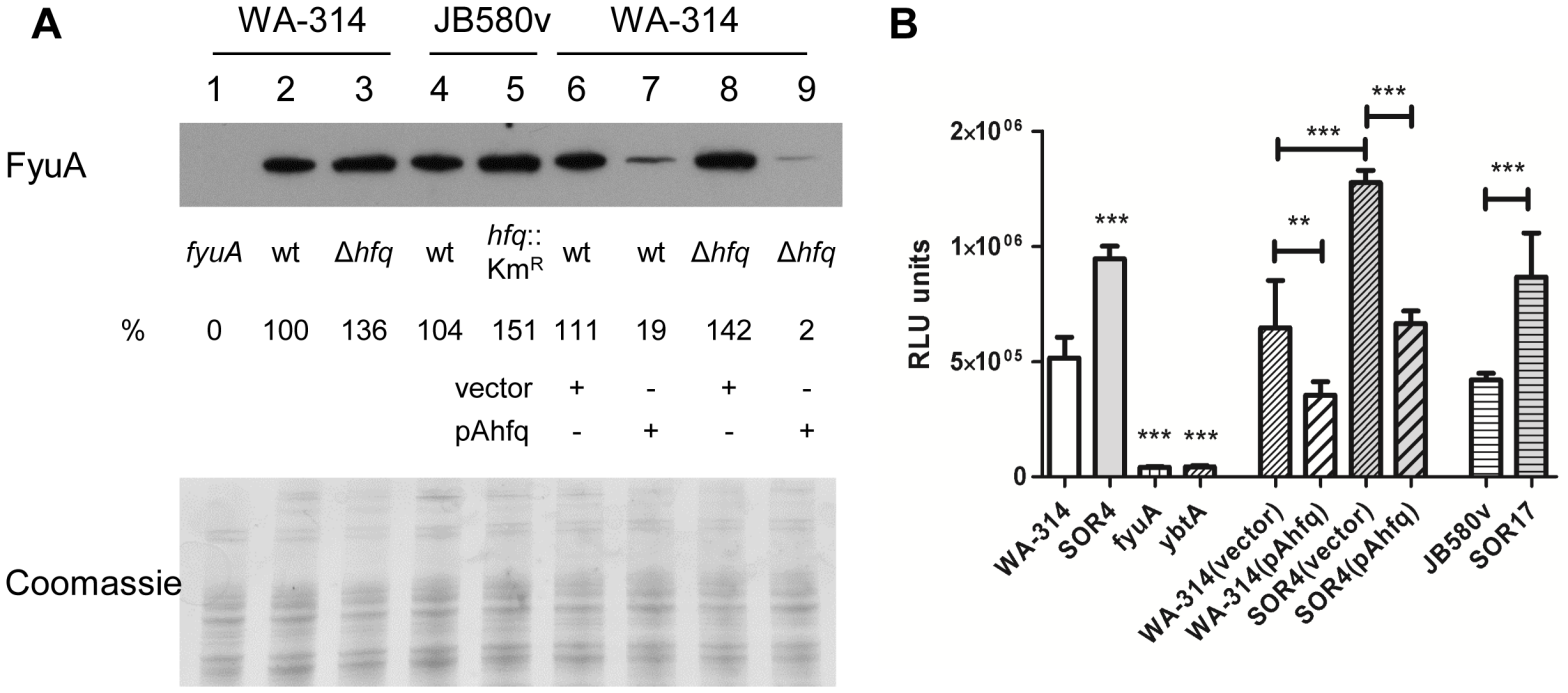
Role of *hfq* in production of yersiniabactin and its receptor FyuA. (A) Immunodetection of FyuA in strains grown for 24 h in LB supplemented with DIP (LBD). Loading was as follows: 1, WA fyuA; 2, WA-314; 3, SOR4; 4, JB580v; 5, SOR17; 6, WA-314(pACYC184ts); 7, WA-314(pAhfq); 8, SOR4(pACYC184ts); and 9, SOR4(pAhfq). Upper panel shows the immunoblot. The relative signal for each band compared to wild type (which was set to 100%) is indicated. Bottom panel shows part of Coomassie blue-stained gel used as loading control. (B) Reporter assay measuring yersiniabactin production. Following growth for 24 h in LBD at 37°C, bacterial culture supernatants were harvested. They were applied to a reporter strain which expresses luciferase in response to yersiniabactin. Luciferase activity was determined after incubation of the reporter strain for 24 h at 37°C. Results are the mean and standard deviation of duplicate cultures each assessed in triplicate. Significance was calculated with Student‘s unpaired *t*-test (**P<0.01; ***P<0.001). Similar results were obtained in three independent experiments.

Next we tested whether Hfq also played a role in FyuA production under low-iron conditions (to alleviate Fur repression). Bacteria were grown for 24 h at 37°C in LB supplemented with the ferrous iron chelator DIP (LBD), and then FyuA was detected by immunoblotting (Fig. 7A). The outer membrane receptor was more abundant in *hfq*-negative strains than in parental strains (ca. 30–50% increase). Most strikingly, increased production of Hfq from plasmid pAhfq led to an 80% reduction in FyuA in the wild type strains. Taken together, our results indicate that Hfq inhibits the production of FyuA.

### Role of hfq in siderophore production

Since the transcriptional regulator YbtA regulates expression of *fyuA* as well as the genes involved in yersiniabactin biosynthesis, we next tested whether Hfq played a role in yersiniabactin production. Using a reporter strain which contains a yersiniabactin-responsive promoter fused to luciferase, we were able to detect yersiniabactin released into the supernatants of bacteria grown in LBD. As shown in Fig. 7B, the *hfq*-negative strains SOR4 and SOR17 produced nearly twice as much siderophore as their respective parental strains. Complementation was achieved by expressing *hfq* from pAhfq (Fig. 7B). Our data indicate that Hfq represses production of the siderophore yersiniabactin in *Y*. *enterocolitica*.

### Role of hfq in type III secretion

**Figure 8:**
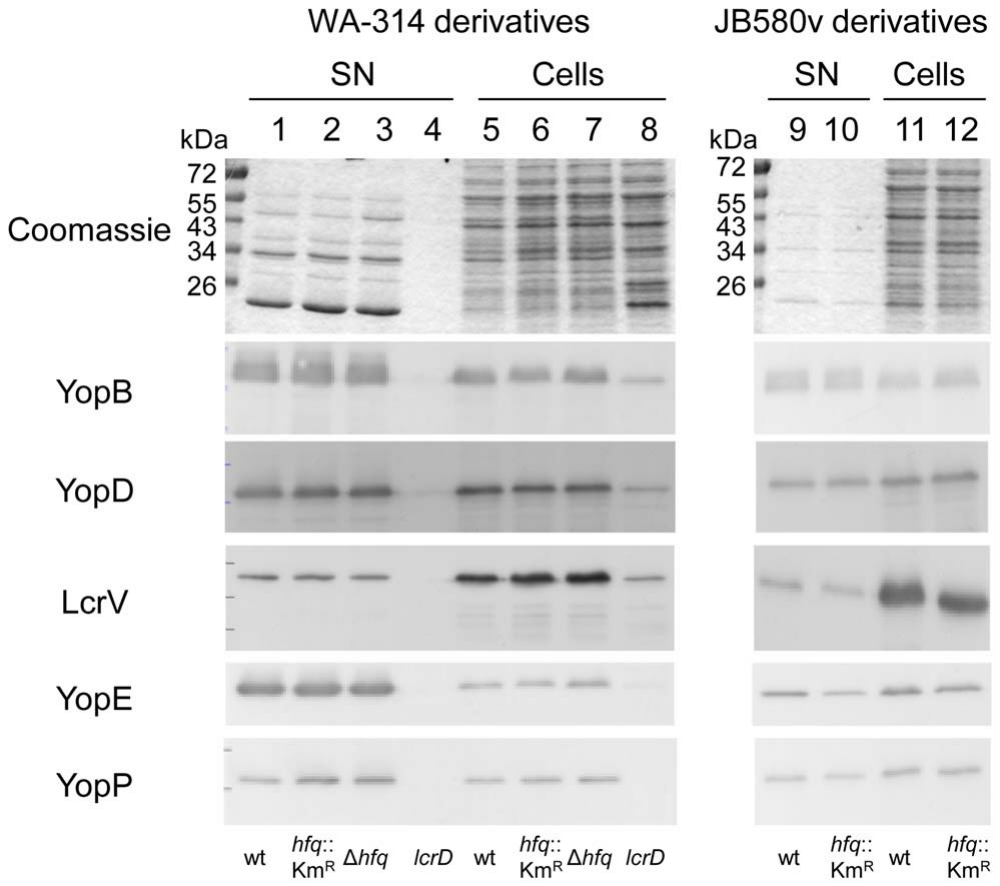
Analysis of Yop proteins secreted by *Y*. *enterocolitica*. Proteins secreted into the supernatant (SN, lanes 1-4, 9–10) and proteins from total bacterial cell extracts (Cells, lanes 5–8, 11–12) were analyzed by Coomassie blue staining (upper panel) and by immunoblotting using antibodies specific for YopB, YopD, LcrV, YopE and YopP. Loading was as follows: molecular weight markers (in kDa); 1 and 5, parental strain WA-314; 2 and 6, *hfq* mutant SOR3; 3 and 7, *hfq* mutant SOR4; 4 and 8, TTSS-defective *lcrD* mutant strain WA-314(pYV-515); 9 and 11, parental strain JB580v; 10 and 12, *hfq-*negative strain SOR17.

Given the essential role of Ysc-T3SS for the pathogenicity of *Y*. *enterocolitica* [6], we next investigated the role of Hfq in protein secretion. Following growth under inducing conditions, i.e. at 37°C in Ca^2+^-depleted media, Yop effector proteins secreted into the supernatant were analyzed by SDS-PAGE and Coomassie blue staining. All mutants were tested on at least four different occasions in either low-Ca^2+^ LB or low-Ca^2+^ BHI, and we observed no major differences between the profile of Yop proteins in the supernatants of *hfq* mutants and those of the parental strains (Fig. 8). Immunoblotting also confirmed that YopB, YopD, LcrV, YopP, YopE, YopM, YopH and YopQ were secreted in comparable amounts by parental strains and *hfq* mutants (Fig. 8 and data not shown). Moreover, the amount of Yops detected in cell lysates was also not influenced by the absence of Hfq (Fig. 8). These results are in contrast with those obtained with *Y*. *pseudotuberculosis*, where Hfq promotes the production of Yops [28], and thus point to some difference in Hfq-mediated regulation of virulence factors between the two enteropathogenic *Y* *ersinia* species. When grown at 37°C in LB or BHI with intrinsic Ca^2+^ levels for 1.5 h (conditions allowing some Yop production but not secretion), strains JB580v and SOR17 also produced comparable amounts of cell-associated YopH (data not shown).

### Production of Hfq in Y. enterocolitica serotype O:8

**Figure 9:**
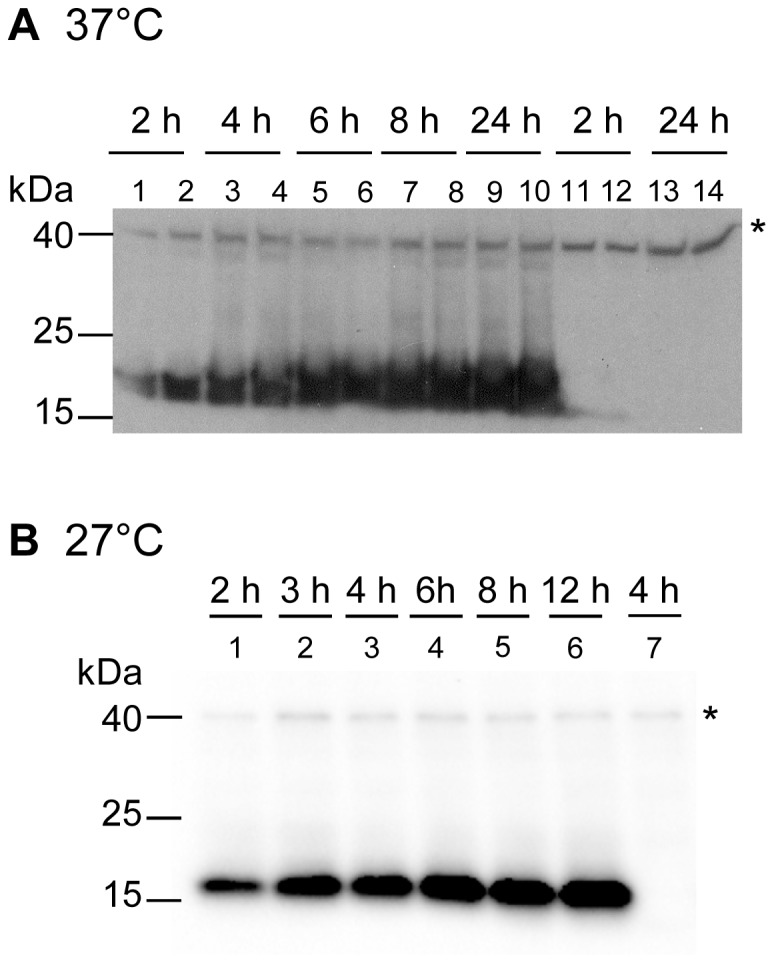
Immunodetection of Hfq-Flag in total protein extracts of *Y*. *enterocolitica*. Time course of expression of Hfq-Flag during growth in LB at 37°C (A) and at 27°C (B). (A) Bacterial extracts of WA^RS^ derivatives (odd-numbered lanes) or JB580v derivatives (even-numbered lanes) were prepared after 2, 4, 6, 8 and 24 h of growth at 37°C. Loading was as follows: extracts from SOR33 in lanes 1, 3, 5, 7 and 9; SOR35 in lanes 2, 4, 6, 8 and 10; parental strain WA^RS^ in lanes 11 and 13; and parental strain JB580v in lanes 12 and 14. The upper band indicated by an asterisk is a background band also present in parental strains WA^RS^ and JB580v (lanes 11–14) and it was used as loading control. (B) Bacterial extracts of JB580v derivatives were prepared after growth for 2, 3, 4, 6, 8 and 12 h at 27°C. Loading was as follows: extracts from SOR35 in lanes 1–6 and parental strain JB580v in lane 7.

To facilitate Hfq detection in *Y*. *enterocolitica* strains, we tagged the chromosomal *hfq* gene with sequences encoding the FLAG epitope to generate strains SOR33 and SOR35. The fusion appears to be functional as both strains exhibited normal growth in LB in contrast to *hfq* mutants (data not shown). Production of Hfq-Flag from plasmid pAhfqFlag was also able to complement the growth of *hfq* mutants (data not shown), confirming that the fusion protein is functional. We next analyzed the time course of production of Hfq-Flag in *Y*. *enterocolitica* grown at 27°C and 37°C in LB in four independent experiments. We observed an increase in the amount of Hfq-Flag in late exponential phase and stationary phase compared to early exponential phase (ranging from 300 to 800% upon growth at 27°C and 300 to 1200% at 37°C) (Fig. 9 and data not shown). Therefore, Hfq-Flag accumulates to higher levels towards the end of exponential phase and beyond.

## Discussion

In this study, we have phenotypically characterized *hfq* mutants in two strains of different lineages of *Y*. *enterocolitica* serotype O:8, strains WA-314 and JB580v. Loss of *hfq* led to the same phenotypes in both strains, indicating that Hfq plays a conserved role in *Y*. *enterocolitica* serotype O:8. We made several observations indicating that the metabolism of *Y*. *enterocolitica* is profoundly influenced by the RNA chaperone Hfq, encompassing the metabolism of carbohydrates, nitrogen, iron and fatty acids, as well as ATP synthesis. In the first step of our analysis we observed that all *hfq* mutants exhibited a slowed growth and entered stationary phase at a lower OD, a phenotype often (but not always) associated with loss of *hfq* in other bacteria, including pathogens [25], [30], [50]. In other pathogenic *Y* *ersinia* spp., inactivation of *hfq* was reported to affect growth to different degrees. *Y*. *pestis* strains lacking *hfq* were most altered in growth [27], [29], especially at 37°C, whereas *Y*. *pseudotuberculosis hfq* mutants had only minor growth defects [28], [29]. Therefore, *Y*. *enterocolitica* appears to have an intermediate phenotype. Moreover, in contrast to *Y*. *pseudotuberculosis* [28], lack of Hfq does not affect Yop production and secretion by the Ysc-T3SS in *Y*. *enterocolitica* serotype O:8 strains. Taken together, our results suggest that Hfq and potential Hfq-associated sRNAs could affect metabolism and regulation of pathogenicity factors differently among the pathogenic *Y* *ersinia* species.

Because of the central role of Hfq in post-transcriptional regulation, deletion of the *hfq* gene results in pleiotropic phenotypes in many bacteria. In *Salmonella enterica* sv. Typhimurium, a mutation in *hfq* leads to differential expression of 20% of all genes [52], [53], whereas in *Y* *. pestis* ca. 6% of all genes were affected [27]. Such a broad regulatory effect may be explained by the impact of Hfq on the regulation of transcriptional regulators, such as sigma factors [25], [54], but also by the high number of mRNAs that interact with Hfq. Indeed, up to 15% of *S*. Typhimurium mRNAs are thought to directly interact with Hfq [53]. The Hfq hexamer is believed to bind mRNAs on the proximal side and sRNAs on its distal side [22]. Two studies have defined a consensus for mRNA sequences bound to Hfq. The first one analyzed the quaternary structure of Hfq bound to RNA and defined a region with four or five (ARN) triplet repeats where R is a purine nucleotide and N any nucleotide [55]. The second study identified a consensus by genomic SELEX: AAYAAYAA, where Y represents pyrimidines (C or U) [56]. An inspection of the genome of *Y*. *enterocolitica* strain 8081 shows that both consensus can be found in 38 annotated mRNAs within 40 nucleotides of the ribosome binding site (preliminary results), suggesting that Hfq might interact directly with these mRNAs and yet unknown sRNAs to regulate their stability and/or translation.

### Carbohydrate metabolism

In this study we observed that Hfq represses carbohydrate metabolism in *Y*. *enterocolitica*. Enzymes associated with glycolysis (PykF) and the pentose phosphate pathway (TktA and TalB) were more abundant in the cellular extracts of the *hfq* mutant. Moreover, we observed increased media acidification upon growth in API-20E wells containing inositol, and upon growth on agar media containing glucose or mannitol. Interestingly, in *Y*. *pestis*, a strain mutated in *hfq* shows an increase in transcripts encoding PykF and MtlK, a putative mannitol transporter, suggesting that Hfq also represses glycolysis and carbohydrate transport in this pathogenic species [27]. In addition, we observed that 1,2-PD utilization (Pdu) is repressed by Hfq: (1) in our 2-DE analysis performed with strains grown overnight at 27°C, five Pdu proteins (PduA-D and PduG) were more abundant in the *hfq*-negative strain, (2) when grown in the presence of 1,2-PD, overexpression of Hfq in wild-type strains led to a decrease in media acidification. 1,2-PD is a by-product of fucose and rhamnose metabolism that is found in the gut. Although 1,2-PD utilization has so far not been investigated in *Y* *. enterocolitica*, it was mainly studied in *S*. Typhymurium where it is believed to promote growth *in vivo* as well as intracellular multiplication in macrophages [57], [58], suggesting metabolic adaptation to niches relevant to pathogenesis. In *S*. Typhimuriun, *pdu* gene expression is controlled by the transcriptional activator PocR [59] and several global regulators, including Hfq [52], [60]. In *Y* *ersinia* spp., *pdu* genes are restricted to a subset of species, and are notably absent from the genomes of *Y* *. pestis* and *Y* *. pseudotuberculosis* [61], [62], suggestive of adaptation to different niches.

### Nitrogen metabolism

Besides carbohydrate metabolism, several proteins involved in nitrogen metabolism were also influenced by Hfq: i.e. OppA, ornithine decarboxylase and tryptophanase. Our 2-DE analysis revealed that *Y*. *enterocolitica* Hfq represses production of OppA, a conserved periplasmic oligopeptide-binding protein. Transcript analysis using microarrays have shown a similar regulation of *oppA* expression by Hfq in *E*. *coli*, *S*. Typhimurium and *Y*. *pestis* [27], [53], [63]. At least one Hfq-dependent sRNA, GcvB, was shown to directly repress OppA production in *E*. *coli* and *S*. Typhimurium [64], [65]. GcvB is conserved in many bacteria, including pathogenic yersiniae [32], [66]. We also observed that ornithine decarboxylase activity is increased in *hfq*-negative strains, suggesting that polyamine synthesis is repressed by Hfq. In *Y*. *pestis*, polyamines are synthesized by arginine and ornithine decarboxylases (SpeA and SpeC, respectively) and are important for biofilm formation [67]. Finally, tryptophanase was another enzyme identified by 2-DE that was more abundant in *Y*. *enterocolitica* strains lacking Hfq, and we observed a corresponding increase in indole production. Increased indole production is also associated with loss of Hfq in *E*. *coli* [68]. Indole serves as an intercellular signalling molecule in bacterial populations, playing an important role in bacterial physiology, biofilm formation, induction of pathogenicity factors and drug resistance [69]. Through its role in promoting entry into stationary phase, increased indole concentrations could explain, at least in part, the lower yield of *Y*. *enterocolitica hfq* mutants in LB, and conversely why the wild-type strains overexpressing Hfq reach higher cell densities. In summary, Hfq inhibits the production of proteins involved in nitrogen metabolism and potentially in biofilm formation.

### Iron metabolism

Our 2-DE analysis identified two siderophore receptors FcuA and FyuA as increased in a *hfq* mutant. The negative effect of Hfq on FyuA production was confirmed using two different assays (pesticin sensitivity and immunoblotting). Moreover, under low iron conditions, we could show that Hfq inhibits yersiniabactin production, the only siderophore known to be produced by *Y*. *enterocolitica*. Taken together our results suggest that iron metabolism is affected by Hfq in this organism. A conserved sRNA, RyhB, has been implicated in controlling iron homeostasis in several enterobacteria [70]. Its expression is induced under low-iron conditions upon relief from the repression of the global ferric uptake regulator Fur. RyhB represses the translation of proteins or enzymes associated with iron, such as the superoxide dismutase or the succinate dehydrogenase. This mechanism leads to an effective increase in levels of free intracellular iron upon iron starvation [70]. In addition to its role in iron sparing, the *E*. *coli* RyhB promotes the production of the siderophore enterobactin by increasing the production of a permease involved in the uptake of a siderophore precursor molecule [71] and by reorienting the amino acid metabolism towards siderophore synthesis [72]. In *P*. *aeruginosa*, siderophore production has not been directly assessed in strains lacking *hfq* or the functional RyhB-like sRNAs, but transcriptome analysis of an *hfq*-negative strain revealed a decrease in the transcripts encoding siderophore biosynthetic genes [73]. To our knowledge (and in contrast to the examples just mentioned) *Y*. *enterocolitica* is so far the only example where Hfq exerts a negative effect on siderophore production. Further investigations will aim to assess whether this effect is mediated through metabolic alterations or through sRNAs specific for genes involved in yersiniabactin biosynthesis, e.g. gene *irp2* whose mRNA carries a putative Hfq-binding motif.

### Resistance to stress

As seen in other bacteria [30], [52], [60], [63], [74], loss of Hfq in *Y*. *enterocolitica* leads to induction of the stress pathways governed by RpoE and RpoH: the 2-DE proteome analysis identified the chaperones ClpB and HtpG and the protease Lon (RpoH regulon) and the periplasmic protease DegP (RpoE regulon) [75]. We also observed that Hfq promotes resistance to oxidative stress, as has been observed in many bacteria, including *Y*. *pseudotuberculosis* and *Y*. *pestis* [25], [27], [28]. Increased sensitivity to hydrogen peroxide in *hfq*-negative *Y*. *enterocolitica* correlated with diminished amounts of AhpC, a putative peroxiredoxin in *Y*. *enterocolitica*. In *Y*. *pestis*, disruption of *hfq* was associated with a decrease in the *katA* transcript that encodes catalase[27]. In addition to its reduced survival to oxidative stress, *Y*. *enterocolitica hfq*-negative strains were also more sensitive to acidic pH. Factors involved in *Y*. *enterocolitica* resistance to acid include RpoS (a conserved target of Hfq-mediated regulation [25]), OmpR and urease [3], [76], [77]. Here, we showed that Hfq promotes urease activity and production of the UreB urease subunit. As previously mentioned urease promotes virulence of *Y*. *enterocolitica*, probably by enhancing bacterial survival at the acidic pH of the stomach [3]. Despite its key role in the early events of host colonization, relatively little is known about factors involved in its regulation in *Y*. *enterocolitica*. Its production is elevated at low pH or at low temperatures in stationary phase but does not depend on the sigma factor RpoS [17]. Here we have identified Hfq as a positive regulator of urease production in *Y*. *enterocolitica*. In *Y*. *pestis*, although urease is inactivated by a point mutation [78], transcripts encoding UreB, UreC and UreE were less abundant in an *hfq* mutant [27], suggesting that the role of Hfq in promoting urease expression has been retained. In *Y*. *enterocolitica*, since the leader transcript encoding UreA, UreB and UreC carries a putative Hfq-binding site (corresponding to both consensus), it is tempting to speculate that Hfq could directly increase the *ureABC* mRNA stability along with a yet unknown sRNA. Alternatively, Hfq could promote production of a regulator of urease genes, e.g. OmpR which is a positive regulator of urease gene transcription in *Y*. *pseudotuberculosis* [79].

### Production of Hfq

In this study we examined the production of chromosomally encoded Hfq-Flag. Growth stage was found to influence the amount of the RNA chaperone in *Y*. *enterocolitica*, with a maximum production in late exponential and/or stationary phase. In *E*. *coli*, the amount of Hfq protein is known to increase in slow-growing bacteria [80], but has been reported to either decrease [81] or increase [82], [83] in stationary phase. In *P*. *aeruginosa*, Hfq levels rise upon entry into stationary phase [73]. The higher amount of Hfq upon entry into stationary phase parallels the observed increase in expression of many sRNAs at this growth stage in some organisms, including *Y*. *pseudotuberculosis* [32]. In light of the role of Hfq-dependent sRNAs in modulating metabolism in enterobacteria [84], it is interesting that Hfq production itself is influenced by global metabolic regulators such as cAMP-dependent catabolic repressor protein CRP and the carbon storage regulator CsrA in *E*. *coli* [85], [86] or the ppGpp-mediated stringent response in *Shigella flexneri* and *S*. Typhimurium [87], [88]. Whether Hfq production in *Y*. *enterocolitica* is influenced by metabolic cues, such as carbon and nitrogen sources for example, will be subject of further investigation.

### Hfq and pathogenicity factors

Our work could demonstrate that Hfq influences the production of two known virulence factors of *Y*. *enterocolitica*, i.e. urease and yersiniabactin. Moreover, our proteomic approach also suggests that Hfq represses the production of the lipopolysaccharide deacylase LpxR/SfpA, which also contributes to pathogenicity [89], [90]. Therefore, with the previously described role of Hfq in enterotoxin Y-ST production [26], a total of four pathogenicity factors are regulated by the RNA chaperone in *Y*. *enterocolitica*.

In summary, we have investigated the scope of Hfq-dependent processes in *Y*. *enterocolitica* and found many phenotypes linked to the loss of the RNA chaperone, such as changes in metabolism, stress resistance and production of pathogenicity factors. Of course many of the regulatory effects described here are likely to be indirect, and may reflect changes associated with RpoE, RpoH or RpoS for example. In addition, the higher production of proteases (Lon, DegP) most probably affects the stability of some proteins. Lon is well known to contribute to the regulation of several pathogenicity factors in Yersiniae, for example through proteolysis of RovA, the transcriptional activator of early virulence genes [20]. Intriguingly, we found that the products of three RovA-repressed genes, i.e. *ompX*, *oppA* and *tnaA* [91], were increased in an *hfq* mutant. We also expect that some of the observed phenotypes are directly mediated by Hfq and its role as a RNA chaperone. Future work will aim to discover Hfq-dependent sRNAs and their role in the physiology and virulence of *Y*. *enterocolitica*.

### Accession to proteomics data

The mass spectrometry proteomics data have been deposited to the ProteomeXchange Consortium (http://proteomecentral.proteomexchange.org) via the PRIDE partner repository [92] with the dataset identifier PXD000475 and DOI 10.6019/PXD000475.
